# Sociodemographic and clinical characteristics of youths and parents seeking psychological treatment for school attendance problems

**DOI:** 10.1371/journal.pone.0261449

**Published:** 2022-01-26

**Authors:** Daniel B. Johnsen, Johanne J. Lomholt, David Heyne, Pia Jeppesen, Morten B. Jensen, Wendy K. Silverman, Mikael Thastum

**Affiliations:** 1 Department of Psychology and Behavioural Sciences, Aarhus University, Aarhus, Denmark; 2 TrygFonden’s Centre for Child Research, Aarhus University, Aarhus, Denmark; 3 Institute of Psychology, Leiden University, Leiden, Netherlands; 4 Faculty of Health and Medical Sciences, Department of Clinical Medicine, University of Copenhagen, Child and Adolescent Mental Health Center, Mental Health Services—Capital Region of Denmark, Copenhagen, Denmark; 5 Department of Economics and Business Economics, Aarhus University, Aarhus, Denmark; 6 Yale University Child Study Center, New Haven, Connecticut, United States of America; Chiba Daigaku, JAPAN

## Abstract

**Background:**

Knowledge of school attendance problems (SAPs) is needed to inform treatments targeting SAPs and protecting youths from negative outcomes associated with SAPs.

**Objectives:**

This study examined the school absence, absence categories (i.e., absence due to illness, excused, non-excused), sociodemographic characteristics, and mental health problems among youths seeking psychological treatment for SAPs.

**Methods:**

The study used a cross-sectional design. Sociodemographic and clinical characteristics of 152 help-seeking youths with SAPs (i.e., >10% absenteeism) and their parents were examined. The data were derived from the baseline assessment conducted before treatment start.

**Results:**

Older youths, youths with mental health problems, and youths whose parents had mental health problems exhibited higher levels of absence. Lower levels of non-excused absence were found among youths with highly educated fathers, and youths living with both parents. Many youths had clinical levels of anxiety, depression, or ‘emotional and behavioral difficulties’.

**Conclusion:**

The study highlights the need for early intervention, addressing a broad range of mental health problems.

**Clinical trial registration:**

ClinicalTrials.gov: NCT03459677.

## Introduction

Absence from school can be problematic for youths, their families, and society in general [1–3]. *School attendance problem* (SAP) refers to difficulty attending school or absence from school that is problematic because of its frequency and/or duration [4–6], but definitional bench-marks for SAPs are still lacking [7]. Every missed school day is a day of missed education and the negative effects are incremental [8]. At the same time, the existence of a specific threshold for defining the presence of a SAP would aid communication among professionals and comparative research. In Denmark, Australia, the UK, and the USA, missing 10% or more of school has been described as concerning or problematic, and the prevalence rates of youths with absence above this threshold range from 11% to 25% [9–12]. Findings from a panel analysis of a Danish community study showed that youths with 10% or more school absence in the last three months, were significantly associated with health-related problems, as well as emotional and behavioral prolems [13].

There are high costs associated with SAPs. School absence has been associated with lower academic achievement among youths [14], higher risk of school drop-out [15], and subsequent unemployment [16]. SAPs have also been linked with health risk behaviors [17] and mental health problems among youths [18,19]. Parental factors associated with SAPs include mental health problems [20] and low education [21]. Because school absence begets future school absence [22], and the costs associated with SAPs are high, there is a need for early detection and intervention [23,24]. To inform early detection and intervention, research into risk and protective factors is needed.

Much research has addressed SAPs as specific categories or types of school absence [4]. For example, studies addressing *school refusal* have focused on school absence related to anxiety [e.g., 25,26] while studies addressing *truancy* have focused on non-excused absence [e.g., 27,28]. Sociodemographic characteristic and mental health problems are often described for distinct subgroups of youths with SAPs [e.g., 19,29], and these have been found to be differentially associated with specific types of absenteeism. For example, non-excused absence (i.e., unauthorized absence without a doctor’s note or other permission from the school) was found to have a greater impact on youths’ academic achievement compared to excused absence [14]. The higher negative impact of non-excused absence on academic achievement might reflect more problems than just time away from school, such as behavioral, family and school engagement issues [2].

In the current study, variables were investigated for their relation to the total amount of school absence. This was done, in part, because even a single day of absence may negatively affects students’ academic achievement regardless of the reason for the youths’ school absence category [2]. Furthermore, because variables related to SAPs have different associations to different types of school absence, all registered absence categories were included and examined in the current study.

The generalizability of knowledge regarding school absenteeism has been hindered by the large variability in how absenteeism has been measured. Self- and parent-reported school absence is commonly used in studies of associations between SAPs and other variables, providing first- and second-hand accounts of absence [30,31]. However, these accounts of school absence might be less accurate when youths or parents are asked to recall absences across a long period of time [32]. In addition, youths might underreport specific categories of absence such as non-excused absence, if there are consequences associated with such absences (e.g., detention or economic sanctions) [33]. The present study used parent-reported school attendance data to identify youths with SAPs, while registry-based attendance data were used to examine the youths’ school absence in the previous academic year.

The use of school attendance registries, to monitor the amount of school absence and registered absence category (e.g., absence due to illness, excused absence, or non-excused absence) at the individual level, is common practice in many school systems [10,12]. Researchers have used registry based data to examine the development of absence over time [2,23], assess different types of SAP based on different absence categories (e.g., Truancy [28]), and to determine the severity of SAPs among youths in treatment studies [26]. However, irregularities and missing registration have been found in registry data [34,35]. In the current study registry-based school attendance data were used to characterize duration (i.e., short- and long-term school absence) and all registered categories of absence.

There is substantial evidence of a relationship between SAPs and mental health problems in youths (Heyne, Kearney, Finning, in press). Among clinical samples of youths with SAPs common mental health problems are anxiety, depression, and behavioral problems [19,20,36]. Studies of SAPs among youths in community samples also reveal associations with anxiety, depression, and behavioral problems [e.g., 31,37,38]. However, few studies linking SAPs with mental health problems used a measure of school absenteeism to identify the presence of a SAP. Rather, they relied on brief statements from participants declaring that the youths had problems attending school [e.g., 19] or they used a measure of motivation for absenteeism but not of actual absenteeism [e.g., 37]. Because absenteeism was not measured, it was not possible to explore relationships between levels of absenteeism and levels of mental health problems. Studies that have reported levels of absenteeism among youths with SAPs were often limited to youths diagnosed with a mental health problem [e.g., 39–41], substantially limiting the extent to which relationships between levels of absenteeism and levels of mental health problems could be studied. The current study permitted a fuller investigation of mental health problems among youths with SAPs according to level of school absenteeism. In the process, the proportion of youths with clinical levels of mental health problems was studied to estimate the frequency of different mental health problems among youths with SAPs, to understand which problems need to be addressed in future SAP interventions.

In the present study of a large help-seeking sample of youths with SAPs, we aimed to: 1) explore levels of absenteeism, with respect to total school absence, and with respect to the absence categories ‘excused absence’, ‘non-excused absence’, and ‘absence due to illness’; 2) describe the sample’s sociodemographic variables and mental health problems in light of youths’ total absence and absence categories; and 3) determine the proportion of youths with SAPs who experience a clinical level of anxiety, depression, or ‘emotional and behavioral difficulties’.

## Materials and methods

### Participants

Data for the present study were drawn from a sample of 152 youths with SAPs, with a mean age of 12.2 years (*SD* = 2.16; age range 6–16). All participants were involved in a treatment study for SAPs (see Thastum et al. [42] for complete details of study methodology) registered at ClinicalTrials.gov: NCT03459677. The inclusion criteria for participation were: (a) youths enrolled in a public school within Aarhus or Odder Municipality, Denmark; (b) aged 6–17 years, (c) more than 10% school absence during the last three months of school based on parent-reported school absence, (d) the holders of the parental rights gave written consent for participation a clinical treatment study. Private schools register students’ school absence differently from public schools, and they are outside the municipality’s jurisdiction, rendering school absence data unavailable. The relatively low-threshold of 10% school absence was chosen to define a SAP, as previous studies have indicated possible negative consequences associated with even lower amounts of school absence [2,8]. The included municipalities comprise of a large and representable section of the Danish population. Aarhus is the second most populated municaplity in Denmark providing a representative sample of youths, primarily in urban and suburban schools. Odder municipality has an close to average population density, and make up youths from suburban and rural schools.

### Procedure

The Department of Psychology and Behavioral Sciences at Aarhus University, where the study was conducted, did not have an institutional review board, so in accordance with Danish procedures, the Central Denmark Regional Ethics Committee was consulted. In keeping with the regulations of the health research committees in Denmark, questionnaire-based studies are often denied access to a full evaluation. However, a study protocol was provided to the Central Denmark Regional Ethics Committees who confirmed, that the project was not encompassed by the term ‘Bio-medical research’ and as such not eligible for Committee review, meaning that the study needed no further approval. The project was registered at the Danish Data Protection Agency (Ref no. 2016-051-000001). Participants were recruited for the study between August 2017 and March 2019. Following inclusion, the youth and one parent or primary caretaker completed a battery of web-based questionnaires. The municipalities provided school attendance records for each youth. Data presented in the current study were derived from the baseline assessment conducted before treatment start.

### Measures

***School absence reported by parents*** was collected prior to inclusion. Parents rated rate how much their child had missed school in the last three months based on the following four categories: ‘Less than 10% (less than six absent days)’, ‘10–20% (approximately 6–12 absent days)’, ‘20–30% (approximately 12–18 absent days)’, ‘30–50% (approximately 18–30 absent days)’, ‘>50% (more than 30 absent days)’, ‘100% (the child has not attended school in the last three months)’. This measure was used as an inclusion criterion to determine if the youths had a SAP.

***School absence based on school attendance records*** for the last academic year (last 200 school days) was obtained from Aarhus and Odder municipality. Absent days were coded dichotomously (1 = *absent*, 0 = *present*) and registered by the schools prospectively day-by-day. All absent days in Danish public schools are registered as one out of three categories: (a) absence due to illness, (b) excused absence, or (c) non-excused absence. Absence due to illness is due to sickness or another functional impairing condition that prevents the student from attending school. Excused absence refers to extraordinary absence granted by the schools (e.g., important family events; vacation outside official school holidays), which is not deemed to have negative consequences for the student. Non-excused school absence is defined as absence where parents fail to inform the school of the reason for the absence or fail to provide a medical certificate if requested by the school in periods of absence due to illness [10].

***Sociodemographic information*** was provided by parents and included youths’ *age*, *sex*, *living situation*, and *previously diagnosed mental health problems*. They also reported *parents’ highest achieved education*, and *parental mental health problems*.

***Youth anxiety*** was measured using the Danish version of the *Spence Children’s Anxiety Scale* (SCAS; [43] rated by youths (SCAS) and parents (SCAS-P). The SCAS and SCAS-P assess symptoms of youths anxiety, consisting of 44 and 38 items respectively. The Danish version of the SCAS and SCAS-P has demonstrated good internal consistency [44]. The current samples Cronbach’s alpha values for the SCAS and SCAS-P was .92, and .92 repectiveley.

***Youth depression*** was measured using the Danish version of the *Mood and Feelings Questionnaire*rated (MFQ; [45] rated by youths (MFQ) and parents (MFQ-P). The MFQ and MFQ-P assess a broad range of youth’s cognitive and vegetative symptoms of depression. The Danish version of the MFQ and MFQ-Parent has demonstrated high internal consistency [46]. The current samples Cronbach’s alpha values for the MFQ and MFQ-Parent was .93, and .93 repectiveley.

***Youth emotional*, *and behavioral difficulties*** were measured using the extended Danish version of the *Strengths and Difficulties Questionnaire* (SDQ; [47] rated by youths (SDQ) and parents (SDQ-P). The SDQ and SDQ-P are brief behavioral screening questionnaires assessing youth’s emotional and behavioral difficulties. Higher scores indicate higher levels of difficulties. The Danish version of the SDQ has shown high internal consistency [48]. The current samples Cronbach’s alpha values for the SDQ and SDQ-P was .80, and .78 repsectiveley.

***Interference of youths problems*** were assessd using the youth and parent version of the SDQ subscale for impact and interference (SDQ-Impact; [47] rated by youths (SDQ-Impact) and parents (SDQ-P-Impact). The SDQ-Impact and SDQ-P-Impact assess youth’s distress and the interference of their problems. Higher scores indicate higher interference and impact. The current samples Cronbach’s alpha values for the SDQ-Impact and SDQ-P-Impact was .76 and .64 repectiveley.

### Data analysis

**Fig 1:**
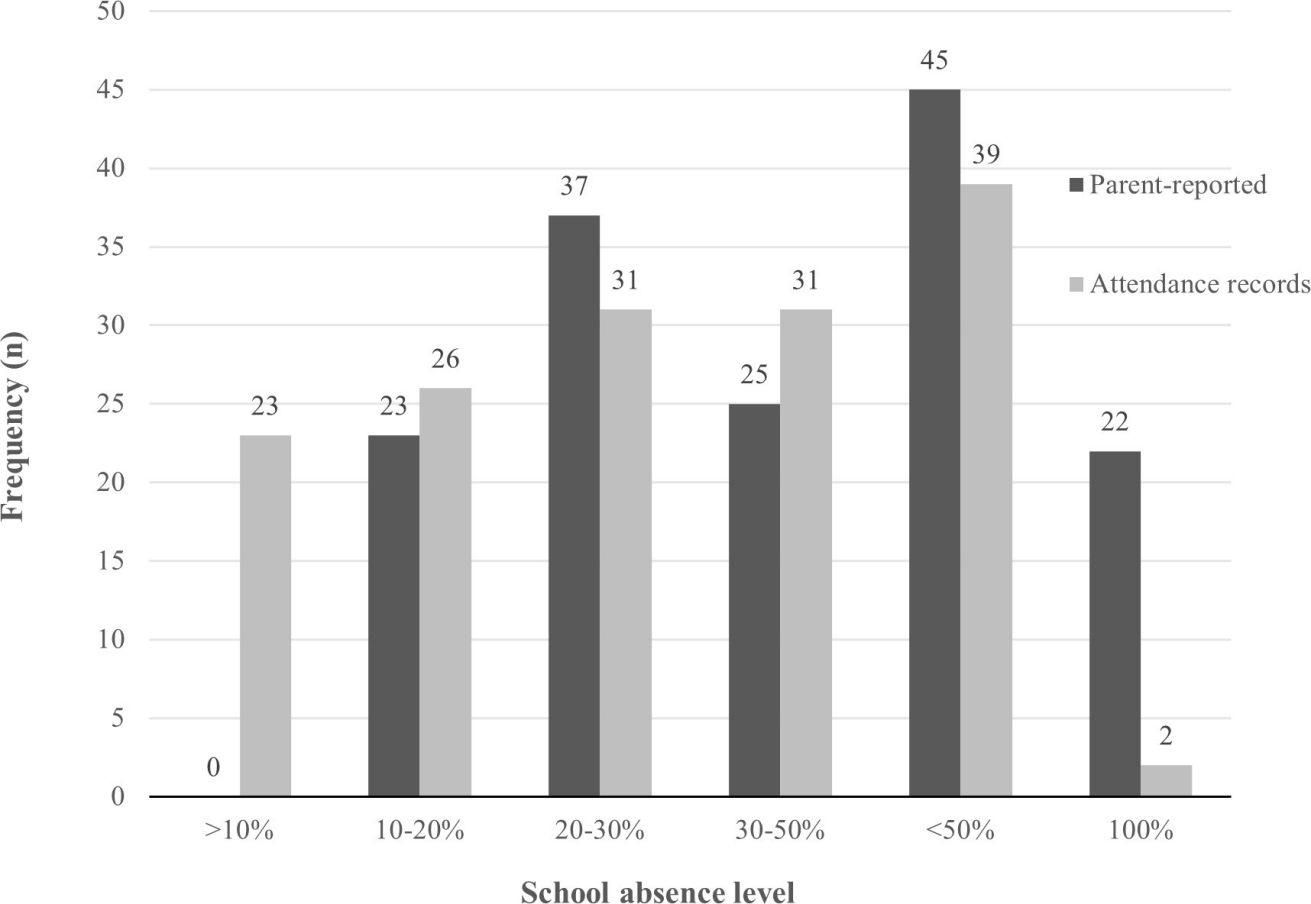
Frequency distribution of youth school absence using parent-reports and attendance records, in the previous three-months of school.

**Fig 2:**
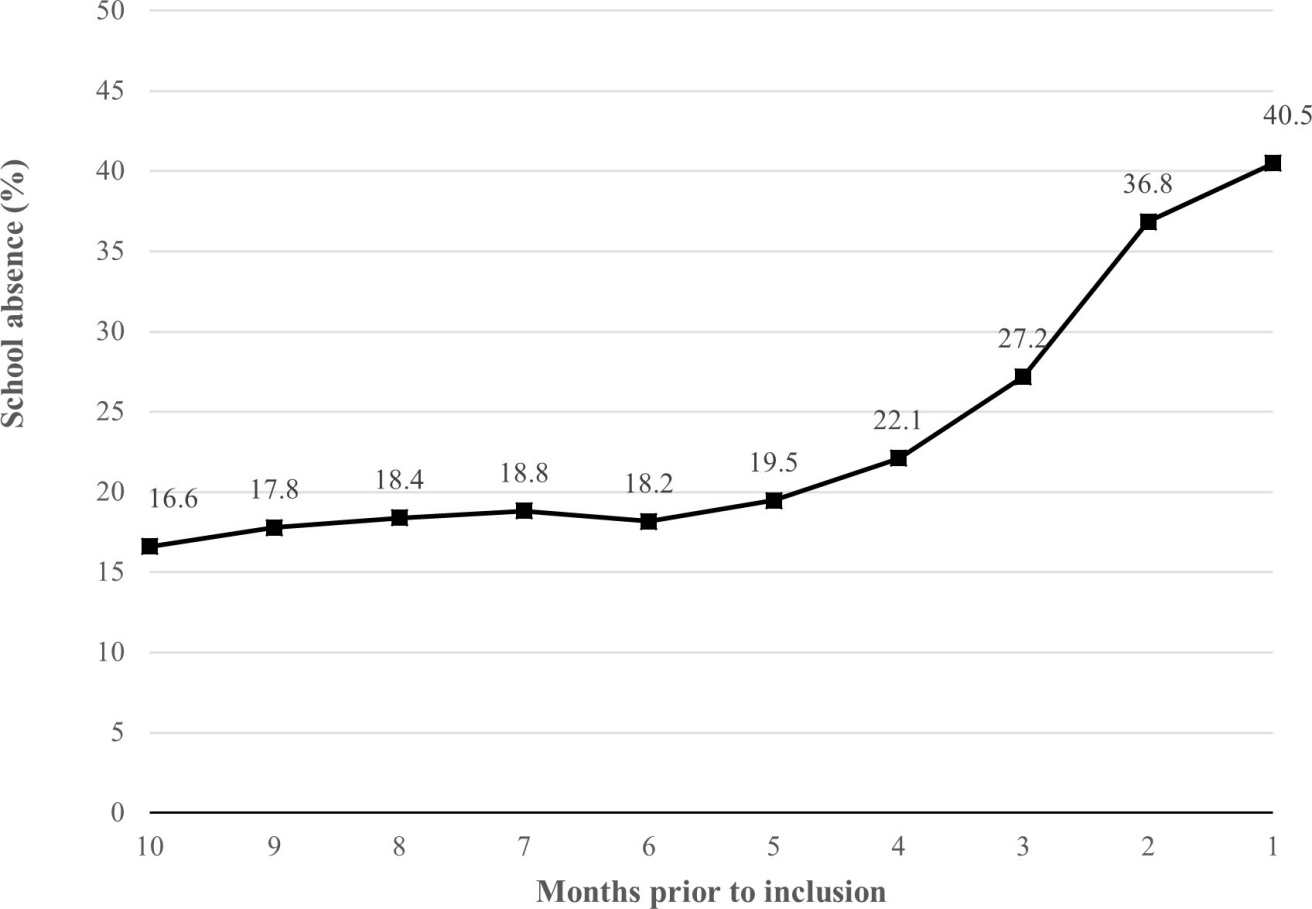
School absence per month (%) in the previous school year.

Using the same categories as the parent-reported school absence (i.e., ‘10%’,‘10–20%’, ‘20–30%’, ‘30–50%’, ‘>50%’, and ‘100%’), we calculated the frequency distribution of the youths school absence based on the attendance records in the previous three months (i.e., 60 school days). Both frequency distrubutions are presented in Fig 1. Based on the school attendance records, we constructed variables for *short-term* and *long-term* school absence. Short-term absence was defined as the percentage of missed school days in the last three months of school (i.e., 60 school days). Long-term absence was defined as the percentage of missed school days in the last 10 months of school (i.e., 200 school days). The mean percentage of each of the 10 school months were also calculated (see Fig 2). The percentage of school absence categorized as either due to illness, excused, or non-excused was calculated, for both the short- and long-term school absence.

The demographic variables relating to youths’ age, living situation, and youths’ and parents’ mental health problems were dichotomized. For age, a group division of ‘6–12 years’ and ‘13–17 years’ was used to reflect the ages of youths in Danish primary and secondary schools. Youths living situation, were divided into either living with both parents or not (i.e., ‘Yes’ or ‘No’). Mental health problems among youths’ were either reported as present or not (i.e., ‘Yes’ or ‘No’), and present or not among one of the parents (i.e., ‘Yes’ or ‘No’). Mothers’ and fathers’ level of education were divided into ordinal variables using three levels of education (i.e., Primary education’ 0–10 years (e.g., primary or secondary school), ‘Secondary education’ 11–15 years (e.g., high school or vocational degree)’ and ‘Tertiary education’ 16–20 years (e.g., masters or doctorate level of education).

Independent samples *t-*tests were used to compare the sample means of school absence and absence categories, divided by youths sex, age, living situation, mental health problems among youths’ and parents’. One-way ANOVA tests were conducted to compare difference in means between different parental levels of education, with post-hoc comparison using Gabriel’s pairwise comparison test.

Participants with elevated scores on the youth- and parent-reported SCAS, MFQ, SDQ, and SDQ-Impact was assessed using Goodman’s [47] recommendations for frequency distribution. Proposing that approximately 80% of a normative community population is in the ‘normal’ range; 10% is in the ‘borderline’ range; and the remaining highest 10% scores are in the ‘clinical’ range. We used Z-scores to calculate cut-off scores for the normal (80%), borderline (10%), and clinical (10%) range, based on means and standard deviation from published Danish community samples on the SCAS, MFQ, and SDQ [44,46,49]. We compared the frequency distribution of elevated scores in the SAP sample with the expected distribution based on the community samples. Chi-squared tests were used to compare the frequencies of ‘normal’, ‘borderline’, and ‘clinical’ range across the SAP and community samples. The comparisons were conducted using age and sex-specific norms provided in the published Danish community samples [44,46,49].

## Results

### School absence

The frequency distribution of both the parent-reported school absence and attendance records provided by the municaplities, are shown in Fig 1. Based on the school attendance records the youths missed on average 34.8% (*SD* = 25.9) of school in their last three months of school, and 23.6% (*SD* = 16.0) in the last academic year. Most of the youths’ absence was registered as due to illness in both the short- (*M* = 56.7%, *SD* = 38.7) and long-term period (*M* = 58.7%, *SD* = 33.4), followed by non-excused (Short-term: *M* = 25.5%, *SD* = 33.7, Long-term: *M* = 24.2%, *SD* = 30.2) and excused absence (Short-term: *M* = 14.6%, *SD* = 26.0, Long-term: *M* = 15.8%, *SD* = 21.2). As shown in Fig 2, there was an increase in school absence throughout the last academic year.

### Mean comparisons of school absence and absence categories

As shown in Table 1, the age-group division showed a significant difference in the amount of long-term absence between the younger (6–12 years, *M* = 20.1%, SD = 12.8) and older youths (13–17 years, *M* = 26.9%, *SD* = 18.0, *t*(150) = -2.73, *p* < .01). There was also a significant difference in long-term absence categorized as non-excused, between the younger (6–12 years, *M* = 19.0%, SD = 26.4) and older youths (13–17 years; *M* = 29.1%, *SD* = 32.8, *t*(150) = -2.10, *p <* .05). Youths’ with mental health problems had a significantly higher percentage of long-term absence (*M* = 29.4%, SD = 19.4) compared to youths without mental health problems (*M* = 21.5%, *SD* = 14.1, *t*(150) = -2.34, *p* <. .01). Youths’ whose parents had mental health problems had a significantly higher percentage of short-term absence (*M* = 41.7%, SD = 27.7) compared to the other youths (*M* = 31.5%, *SD* = 24.4, *t*(150) = -2.30, *p <* .05). There was also a significant difference in the amount of long-term absence between the youths whose parents had mental health problems (*M* = 29.8%, SD = 19.2) compared with the youths who did not (*M* = 20.5%, *SD* = 13.3, *t*(150) = -3.08, *p <* .01). Youths living with both parents had a significantly lower percentage of short-term absence categorized as non-excused (*M* = 19.0%, SD = 31.4) compared to youths in other living situations (*M* = 33.4%, *SD* = 34.9, *t*(150) = 2.67, *p <* .01). The percentage of long-term non-excused absence was also significantly lower among youth living with both parents (*M* = 17.1%, SD = 25.5) compared to youths in other living situations (*M* = 32.9%, *SD* = 33.2, *t*(150) = 3.32, *p <* .01). The percentage of long-term excused absence was significantly higher among youths living with both parents (*M* = 19.2, SD = 25.0) compare to youths in other living situations (*M* = 11.6%, *SD* = 14.2, *t*(150) = -2.34, *p <* .05).

**Table 1:** Mean comparisons of short- and long-term school absence (%) and related absence categories (%).

Short-term absence:		Total absence (%)			Absence due to illness (%)			Excused absence (%)			Non-excused absence (%)		
*Variable*	*Group (n)*	*M (SD)*	*t / F*	*M diff*. *CI*	*M (SD)*	*t / F*	*M diff*. *CI*	*M (SD)*	*t / F*	*M diff*. *CI*	*M (SD)*	*t / F*	*M diff*. *CI*
Sex	*Males (92)*	33.35 (25.69)	-.880	-12.29, 4.72	56.58 (37.32)	-.036	-12.96, 12.50	13.01 (23.13)	-.870	-13.00, 5.08	27.15 (33.23)	.763	-6.78, 15.32
	*Females (60)*	37.14 (26.31)			56.81 (41.01)			16.97 (29.93)			22.88 (34.44)		
Age (years)	*6–12 (74)*	31.69 (24.47)	-1.468	-14.43, 2.13	58.95 (37.04)	.704	-8.00, 16.85	16.65 (27.38)	.957	-4.30, 12.38	21.70 (29.74)	-1.352	-18.03, 3.38
	*13–17 (78)*	37.84 (27.04)			54.52 (40.31)			12.61 (24.64)			29.03 (36.84)		
Mental health problem, Y	*No (112)*	33.71 (24.02)	-0.808	-15.08, 6.40	56.24 (38.46)	-0.230	-15.77, 12.48	14.35 (25.84)	-0.182	-10.37, 8.62	27.63 (34.70)	1.331	-3.99, 20.45
	*Yes (40)*	38.04 (30.73)			57.88 (39.79)			15.22 (26.76)			19.40 (30.16)		
Mental health problem, P	*No (102)*	31.50 (24.44)	-2.304*	-18.88, -1.45	57.15 (39.17)	0.214	-11.81, 14.67	15.75 (27.46)	0.794	-5.31, 12.45	24.16 (33.45)	-0.677	-15.45, 7.56
	*Yes (50)*	41.67 (27.72)			55.71 (38.05)			12.18 (22.82)			28.11 (34.27)		
Living with both parents	*No (68)*	38.38 (24.68)	1.520	-1.92, 14.72	52.91 (37.96)	-1.079	-19.27, 5.66	10.74 (19.83)	-1.714	-14.94, 1.06	33.40 (34.92)	2.670**	3.74, 25.01
	*Yes (84)*	31.98 (26.68)			59.72 (39.23)			17.68 (29.85)			19.03 (31.36)		
Mothers level of education (years)	*0–10 (8)*	35.42 (23.57)	0.907		41.87 (38.73)	2.436		18.74 (20.50)	0.144		39.39 (25.84)	1.130	
	*11–15 (105)*	36.59 (25.97)			61.20 (37.50)			14.70 (27.22)			23.15 (32.86)		
	*16–20 (39)*	30.04 (26.26)			47.54 (40.43)			13.38 (24.00)			28.83 (36.84)		
Fathers level of education (years)	*0–10 (26)*	37.95 (24.59)	0.276		45.79 (40.61)	1.278		7.89 (14.31)	1.408		42.48 (37.26)	5.248**	
	*11–15 (87)*	34.71 (26.19)			58.33 (37.41)			14.64 (25.93)			24.73 (33.38)		
	*16–20 (39)*	33.08 (26.63)			60.23 (39.95)			18.90 (31.31)			15.74 (27.78)		
**Long-term absence:**		**Total absence (%)**			**Absence due to illness (%)**			**Excused absence (%)**			**Non-excused absence (%)**		
Sex	*Males (92)*	23.01 (16.16)	-.550	-6.72, 3.80	57.99 (32.28)	-.321	-12.78, 9.20	16.06 (20.69)	.183	-6.32, 7.61	24.86 (29.29)	.343	-8.20, 11.65
	*Females (60)*	24.48 (15.86)			59.78 (35.36)			15.42 (22.04)			23.14 (31.70)		
Age (years)	*6–12 (74)*	20.06 (12.80)	-2.725**	-11.86, -1.89	63.01 (32.19)	1.555	-2.27, 19.06	16.62 (21.77)	.461	-5.21, 8.39	19.02 (26.38)	-2.088*	-19.57, -0.54
	*13–17 (78)*	26.94 (18.00)			54.61 (34.26)			15.03 (20.68)			29.08 (32.80)		
Mental health problem, Y	*No (112)*	21.53 (14.14)	-2.335**	-14.53, -1.11	59.11 (32.77)	0.251	-10.65, 13.76	15.63 (20.87)	-0.176	-8.41, 7.04	24.38 (30.18)	0.133	-10.28, 11.76
	*Yes (40)*	29.35 (19.42)			57.55 (35.60)			16.31 (22.23)			23.64 (30.52)		
Mental health problem, P	*No (102)*	20.53 (13.25)	-3.079**	-15.30, -3.28	59.41 (33.03)	0.372	-9.28, 13.59	17.47 (22.66)	1.521	-1.53, 11.67	22.14 (28.73)	-1.192	-16.48, 4.08
	*Yes (50)*	29.82 (19.20)			57.25 (34.51)			12.40 (17.43)			28.34 (32.83)		
Living with both parents	*No (68)*	26.29 (14.82)	1.885	-0.23, 10.00	53.97 (33.94)	-1.576	-19.27, 2.17	11.63 (14.21)	-2.341*	-13.94, -1.17	32.93 (33.23)	3.231**	6.13, 25.52
	*Yes (84)*	21.40 (16.68)			62.52 (32.70)			19.19 (25.02)			17.10 (25.52)		
Mothers level of education (years)	*0–10 (8)*	27.63 (17.43)	2.420		43.17 (30.71)	3.008		17.13 (12.89)	0.447		39.69 (26.90)	1.344	
	*11–15 (105)*	25.03 (16.08)			62.97 (32.64)			14.73 (21.08)			22.30 (29.90)		
	*16–20 (39)*	18.87 (14.92)			50.37 (34.30)			18.43 (22.85)			26.07 (31.20)		
Fathers level of education (years)	*0–10 (26)*	28.88 (15.28)	1.736		48.66 (35.15)	1.497		9.08 (11.03)	4.128*		42.26 (34.16)	7.062**	
	*11–15 (87)*	22.43 (15.64)			61.53 (32.06)			14.44 (18.77)			22.88 (29.00)		
	*16–20 (39)*	22.65 (16.96)			59.06 (34.83)			23.34 (28.44)			15.04 (25.18)		

Note: Y: Youths, P: Parents, M: Mean. SD: Standard Deviation. M diff. CI = Mean difference confidence intervals (95%). t: t-value from independent samples t-test, F: F-value from One-way ANOVA.

* p < .05 level (2-tailed).

** p < .01 level (2-tailed).

Regarding parents’ education, there was a significant difference between fathers level of education and short-term non-excused absence (*F*(2, 149) = 5.25, *p* < .01), as well as long-term excused (*F*(2, 149) = 4.13, *p* < .05) and non-excused absence (*F*(2, 149) = 7.062, *p* < .01). Post hoc analysis showed that youths whose fathers’ had only completed a primary level of education had significantly higher levels of short-term non-excused absence compared to those with a secondary (*p* <. 05, 95% CI [0.8, 34.7]) or tertiary (*p* <. 05, 95% CI [6.8, 46.7]) level of education. Similar findings were found for the long-term non-excused absence, showing significant higher percentages in the group only completing a primary education compared to the groups with a secondary (*p* <. 05, 95% CI [4.4, 34.4]) or a tertiary (*p* <. 05, 95% CI [9.6, 44.9]) level of education. Youths whose fathers had completed a tertiary level of education had significantly higher mean percentage of long-term excused absence, compared to those with only a primary level of education (*p* <. 05, 95% CI [1.6, 26.9]).

### Elevated scores on SCAS, MFQ, and SDQ

As shown in Table 2, there was a significant difference between the SAP and community sample in the distribution of youth- and parent-rated scores within the normal, borderline, and clinical range for the total scores on SCAS, MFQ, SDQ, and the SDQ-Impact. The proportion of youths with SAPs scoring within the normal range was lower than the expected distribution on all measures, while the proportion of youths scoring within the clinical range was higher than the expected distribution on all measures. The proportion of youths scoring within the borderline range was close to the expected distribution, except for a higher number of youths in the SCAS for males, and a lower proportion of youths on the SDQ-Impact and SDQ-P-Impact for males.

**Table 2:** Participants with elevated scores on SCAS, MFQ, SDQ-Total and SDQ-Impact, compared with the expected distribution from norm data.

			Youth				Parent			
			SAP	Community^a^	Test statistics		SAP	Community^a^	Test statistics	
			*n (%)*	*n (%)*	*Z-test*	*χ*^*2*^/*p-value*	*n (%)*	*n (%)*	*Z-test*	*χ*^*2*^/*p-value*
SCAS	Males	Normal	39 (42.4)	391 (80)	-7.538*	χ^2^(2) = 2016.24 *p* = < .01	28 (30.4)	210 (80)	-8.741*	χ^2^(2) = 4087.04 *p* = < .01
		Borderline	16 (17.4)	49 (10)	2.058*		10 (10.9)	26 (10)	0.258	
		Clinical	37 (40.2)	49 (10)	7.483*		54 (58.7)	26 (10)	9.623*	
	Females	Normal	21 (35.0)	386 (80)	-7.574*	χ^2^(2) = 1406.00 *p* = < .01	10 (16.7)	220 (80)	-9.582*	χ^2^(2) = 3224.00 *p* = < .01
		Borderline	7 (11.7)	48 (10)	0.419		2 (3.3)	28 (10)	-1.683	
		Clinical	32 (53.3)	48 (10)	10.966*		48 (80.0)	28 (10)	11.700*	
MFQ	Males	Normal	55 (59.8)	364 (80)	-4.133*	χ^2^(2) = 699.44 *p* = < .01	20 (21.7)	274 (80)	-10.633*	χ^2^(2) = 5877.44 *p* = < .01
		Borderline	9 (9.8)	45 (10)	-0.025		8 (8.7)	34 (10)	-0.359	
		Clinical	28 (30.4)	45 (10)	5.296*		64 (69.6)	34 (10)	12.142*	
	Females	Normal	28 (46.7)	428 (80)	-5.750*	χ^2^(2) = 800.00 *p* = < .01	15 (25.0)	289 (80)	-8.815*	χ^2^(2) = 2318.00 *p* = < .01
		Borderline	6 (10.0)	53 (10)	0.028		4 (6.7)	36 (10)	-0.809	
		Clinical	26 (43.3)	53 (10)	7.245*		41 (68.3)	36 (10)	10.829*	
SDQ	Males	Normal	48 (52.2)	623 (80)	-5.996*	χ^2^(2) = 1321.04 *p* = < .01	24 (26.1)	1264 (80)	-11.951*	χ^2^(2) = 4940.24 *p* = < .01
		Borderline	9 (9.8)	78 (10)	-0.070		9 (9.8)	158 (10)	-0.068	
		Clinical	35 (38.0)	78 (10)	7.567*		59 (64.1)	158 (10)	15.018*	
	Females	Normal	23 (38.3)	664 (80)	-7.428*	χ^2^(2) = 1118.00 *p* = < .01	15 (25.0)	1253 (80)	-10.092*	χ^2^(2) = 2054.00 *p* = < .01
		Borderline	9 (15.0)	83 (10)	1.229		8 (13.3)	157 (10)	0.833	
		Clinical	28 (46.7)	83 (10)	8.301*		37 (61.7)	157 (10)	12.110*	
SDQ-Impact	Males	Normal	49 (53.3)	614 (80)	-5.756*	χ^2^(2) = 1832.24 *p* = < .01	22 (23.9)	1264 (80)	-12.411*	χ^2^(2) = 6171.84 *p* = < .01
		Borderline	0 (0)	77 (10)	-3.183*		2 (2.2)	158 (10)	-2.481*	
		Clinical	43 (46.7)	77 (10)	9.604*		68 (73.9)	158 (10)	17.430*	
	Females	Normal	20 (33.3)	652 (80)	-8.265*	χ^2^(2) = 1754.00 *p* = < .01	11 (18.3)	1253 (80)	-11.271*	χ^2^(2) = 3066.00 *p* = < .01
		Borderline	3 (5.0)	82 (10)	-1.278		2 (3.3)	157 (10)	-1.713	
		Clinical	37 (61.7)	82 (10)	11.254*		47 (78.3)	157 (10)	15.676*	

Note: SCAS: Spence Children’s Anxiety Scale, MFQ: Mood and Feelings Questionnaire, SDQ-Total: Strength and Difficulties Questionnaire.

^a^Frequency distribution for the community sample on SCAS, MFQ, and SDQ, was calculated using published samples (44,46,49).

* p < .05 level (2-tailed).

The number of youths scoring within the clinical range were as follows: On the SCAS, 37 (40.2%) males and 32 (53.3%) females. On the SCAS-P, 54 (58.7%) males and 48 (80.0%) females. On the MFQ, 28 (30.4%) males and 26 (43.3%) females. On the MFQ-P, 64 (69.6%) males and 41 (68.3%) females. On the SDQ, 35 (38.0%) males and 28 (46.7%) females. On the SDQ-P, 59 (64.1%) males and 37 (61.7%) females. On the SDQ-Impact score, 43 (46.7%) males and 37 (61.7%) females. Finally, on the SDQ-P-Impact score, 68 (73.9%) males and 47 (78.3%) females.

The majority of the sample were rated within the clinical range of at least one of the total scores of SCAS, MFQ and SDQ, by the youths’ (*n* = 92, 60.5%) or the parents’ (*n* = 132, 86.8%). Among the youths, 31 (20.4%) were within the clinical range of only one measure, and 61 (40.1%) rated within the clinical range of two or more measures. The parents rated 24 (15.8%) youths within the clinical range of only one measures, and 108 (71.1%) youths within two or more measures.

## Discussion

This investigation of a large sample of youths with SAPs found significantly higher levels of long-term school absence among older youths and youths with mental health problems. The older youths also had significantly higher levels of non-excused school absence. Short- and long-term absence were significantly higher among youths whose parents reported having mental health problems. Youths living with both parents had significantly lower levels of short- and long-term non-excused absence, and significantly higher levels of long-term excused absence. Furthermore, youths with highly educated fathers had significantly lower levels of short- and long-term unexcused school absence, and significantly higher levels of long-term excused absence. The study also showed that the majority of youths had a clinical level of symptoms associated with anxiety, depression, or ‘emotional and behavioral difficulties’. Finally, the level of interference caused by the youths’ problems was often in the clinical range.

The average amount of school absence among youths (34.9% short-term; 23.6% long-term) was lower than that reported in previous studies of treatment for SAPs (40 to 80%; [26,41,50]. This might be explained by the fact that in the current study absenteeism was measured across a longer period of time (i.e., 60 and 200 days versus 10 to 20 days in previous studies). Still many youth in the current study displayed problematic absenteeism. On average, they missed more than a month of school in the previous three months, and the percentage of absence across the previous academic year was more than four times the national average (5.8% absence across 200 days) [51].

The youths’ school absences increased during the academic year prior to entry in the study. This development was in line with previous research showing that school absence among youths increases steadily across the school years [2,23]. It was also found that there was a small increase in average monthly absence (5.5%) in the first seven months, while there was a considerably higher increase in absence (18.4%) in the three months prior to inclusion in the study. This suggests that school absence among youths with a SAP increases, and that severe levels of school absence can manifest in a short time. The relatively rapid increase in school absence points to a narrow window of opportunity for early intervention. This resonates with previous calls for early interventions to prevent the escalation of absences into SAPs [7,52], and it emphazises the need of a reliable system for detecting emerging SAPs [e.g., 53].

We discovered discrepancies between the frequency distribution of parent-reported absence data and the registry-based data. According to the attendance records, 23 participants were absent from school less than 10% of the time in the last three months. Of these 23 participants, parents reported them to be absent 10–20% (*n* = 6), 20–30% (*n* = 5), 30–50% (*n* = 4), < 50% (*n* = 7), and 100% (*n* = 1). In addition, we observed that 22 of the 152 participants had 100% absence as reported by their parents, while only two participants had 100% absence based on the attendance records. One explanation for these discrepancies may be that parents over-report youths’ school absence, and another may be that the attendance records underestimate or falsely report school absence [33]. A further explanation for the observed discrepancies could be related to how schools register students’ absence. According to Danish law, public schools are obliged to register students’ days of absence and not their days of attendance [10]. Consequently, when an absent student is not registered as absent, he or she will be automatically registered as having attended school, possibly deflating the number of absences among youths. The identified discrepancies between absence as reported by parents and absence from municipality attendance records raises issues regarding the reliability and validity of attendance records in general, and Danish attendance records in particular.

The present study found that the older youths (12–17 years) had significantly higher levels of total long-term school absence compared to the younger youths (6–12 years). These findings were in line with previous studies showing that older youths have higher levels of school absence and that higher age is a significant risk factor for developing SAPs and later dropout [23,54]. Furthermore, we found that the older youths had significantly higher levels of non-excused school absence compared to the younger youths. These results resemble previous findings showing that non-excused absences (e.g., truancy) were more prevalent among older youths [27].

Regarding sex, no significant differences were found for amount of school absence or absence category. These findings are consistent with Danish national data showing similar levels of school absence for males and females [51]. However, a small majority of the current sample consisted of males (60%), and there were some notable sex differences in ratings on symptoms of anxiety and behavioral problems. Specifically, females reported more symptoms of anxiety (SCAS) and higher interference (SDQ-Impact) than the males, while males reported more behavioral problems (SDQ subscales for Conduct Problems and Hyperactivity/Inattention, see S1 Table). These results are in line with previous findings showing that anxiety disorders are more prevalent among females while behavioral disorders are more prevalent among males [55]. Because symptoms of anxiety (e.g., shyness; avoiding social situations at school) are more likely to go undetected compared with behavioral problems (e.g., disturbing the class; conflict with peers), males may be more likely to be referred for SAPs, possibly explaining the trend observed in the current study.

There was no significant difference in the amount of school absence among youths living with both parents and youths in other living arrangements. However, when analyzing absence by categories, we found that youths living with both parents had less non-excused absence and more excused absence. Although the difference was non-significant, youths living with both parents had lower levels of school absence, lending partial support to previous studies finding a link between youths with separated or divorced parents and SAPs [19,25]. Our findings suggest that in families where both parents are living together, parents are better at reporting the reason for the youth’s school absence to the schools, possibly reducing the prevalence of non-excused absence relative to excused absence.

There were no significant differences in the total amount of school absence in relation to parental education. However, there was a significant difference regarding the percentage of absence categorized as excused and non-excused. Youths whose fathers completed secondary or tertiary levels of education showed a significantly lower amount of non-excused school absence and higher levels of excused absence, compared to fathers completing only a primary level of education. The same tendency was observed for mothers’ level of education, although non-significant. Previous research has identified low parental education as a risk factor for developing SAPs and later school dropout [54]. Perhaps parents with higher education are more successful at acquiring excused leaves of absence for their child (e.g., vacation outside the official school holidays). In any case, the differences in percentage of excused and non-excused absence related to fathers’ education is an important finding. Extensive amounts of non-excused school absence might lead to economic sanctions (e.g., in the UK and Denmark; [10,56]). Our results suggest that economic sanctions are more likely to occur in families where parents have lower levels of education. This could lead to further socioeconomic disparities, because lower levels of education have been linked to low-income families [57].

We found that school absence was higher among youths with a parent who had a mental health problem. Similar results have been reported in previous studies. McShane et al. [19] found a high prevalence of mental health problems among mothers (53%) and fathers (34%) of youths with anxiety based SAPs, and Gubbels et al. [54] reported that parental mental health problems were a significant risk factor for developing SAPs. Our findings suggest that the level of school absence is negatively affected by parental mental health problems. A possible explanation could be that parental mental health problems magnify the challenges parents face when helping a child with a SAP, as suggested by Heyne [58].

Youths diagnosed with a mental health problem had significantly higher levels of long-term school absence compared to youths without mental health problems. These results are in line with previous findings linking mental health problems with SAPs [18,37]. However, no significant difference in the levels of short-term school absence was found between youths with and without a diagnosed mental health problem.

A large proportion of the current sample had scores in the clinical range for anxiety (SCAS), depression (MFQ), or ‘emotional and behavioral difficulties’ (SDQ). There was also a high number of youths who were in the clinical range on more than one of the measures (i.e., SCAS, MFQ, and SDQ). The number of youths presenting with symptoms in the clinical range exceeded that found among youths with SAPs in a community study in the US [18]. Furthermore, our findings indicate that there were more youths with clinical symptoms of anxiety, depression, and ‘emotional and behavioral difficulties’ (youth-reported: 60.5%, parent-reported: 86.8%) compared to the number of youths diagnosed with any mental health problem (26.3%) prior to inclusion. Many youths with SAPs may present symptoms of mental health problems that could go undetected by schools and mental health professionals. Furthermore, the frequency and diversity of symptoms of poor mental health observed among youths indicates the need for SAP treatments designed to address different mental health problems. Previous treatments accounting for comorbidity when treating youths with SAPs have shown promising results in both alleviating symptoms of mental health problems and increasing school attendance [34,35,40,59].

### Strengths and limitations

To the best of our knowledge, this is the first study of school absence among youths with SAPs to use detailed attendance records, enabling description of the youths’ long- and short-term school absence and the categories of absence. Furthermore, the study included two measures of school absence, a parent-reported measure and registry-based attendance records. Three limitations of the study are noteworthy. First, because the sample was limited to families who were receptive to intervention for SAPs, the findings cannot be generalized to families who do not seek treatment despite the presence of a child with a SAP. Some of the families who do not seek treatment may represent cases of *school withdrawal*, which is a type of SAP characterized by parents willfully keeping their child at home or exerting little effort to get their child to attend school [4]. These parents are unlikely to refer themselves to a program for the treatment of SAPs. The characteristics of youths and parents for whom *school withdrawal* applies remain understudied. Second, the study is cross-sectional and therefore no causal inferences can be drawn. For example, it remains unclear as to whether the increase in youths’ school absence precedes the development of symptoms of mental health problems, or if the development of mental health problems contributes to a subsequent increase in school absence. Third, the comparison of elevated scores on SCAS, MFQ, SDQ, and SDQ-Impact are conducted using previously published Danish community samples. Although the studies include large samples, the scores from these community samples might not be generalizable to the Danish population.

### Scientific and practical implications

Several implications arise from the current study. The level of school absence was found to increase during the previous academic year, and rapidly in the three months prior to inclusion, highlighting the need to address school absence early to prevent a further increase in absence over time. Youths with mental health problems were likely to have higher levels of school absence in the previous academic year. Mental health problems were also prevalent for both mothers and fathers, and youths who had at least one parent reporting mental health problems were more likely to have higher levels of long- and short-term school absence. This suggests that when SAPs have been identified among youths, they should be screened for mental health problems and information regarding parents’ mental health problems should be gathered. The high proportion of youths with clinical levels of one or more different mental health problems highlights the need for SAPs interventions that can encompass complex and comorbid mental health problems. Economic sanctions related to non-excused absence should be used with caution, as sanctions are more likely to affect low-income families and possibly lead to larger socioeconomic disparities in society. By using high thresholds of school absence to identify SAPs among youths, we risk overlooking youths struggling with mental health problems. In a recent study conducting a panel analysis of the risk factors and consequences of school absence, researchers showed that youths with absence above 10% in a one- or three-month period, were more likely to have cognitive handicaps, developmental disorders, as well as the use of psychopharmacological medication, compared to youths with absence between 5–10% of a month [13]. Our results further support these findings, and the high proportion of youths with clinical levels of mental health problems in the present sample, indicate that the utilization of a relatively low-threshold of 10% school absence is an approporiate threshold for identifying SAPs. The identified discrepancies between school absence reported by parents and attendance records highlight the need more valid and reliable systems for measuring school absence.

## Conclusions

What were the characteristics of a help-seeking sample of youths with SAPs? Youth and parent reports showed that the sample consisted of youths with high levels of school absence, high levels of emotional and behavioral symptoms, and considerable impact on their functioning. The youths’ school absence increased during the previous academic year, with a rapid increase in the three months prior to entering the study. Older youths and youths diagnosed with a mental health problem had high levels of long-term school absence. Youths living with both parents had less non-excused absence and more excused absence. In families where at least one parent had a mental health problem, youths were more often missing school. Youths from families where fathers had completed a high level of education had lower levels of school absence categorized as non-excused. The majority of the sample had symptoms of mental health problems within a clinical range. Discrepancies were found between the parent-reported and registry-based data on school absence, indicating that registry based attendance records are not failsafe data sources. Future studies should examine the reliability and validity of data from school attendance records. Efforts need to be made to improve the accuracy of registration systems in schools. Finally, it would seem that interventions that account for the complexity of SAPs [e.g., 34,35,40,59] are more likely to be effective for youths presenting high levels of school absence together with a complex clinical presentation including anxiety, depression, and/or behavioral disorders.

## Supporting information

S1 Table Mean and standard deviations from youth and parent ratings on all total scales and sub-scales of SCAS, MFQ, and SDQ grouped by gender and age group. (PDF)
