# Supplementary material for: Sociodemographic and clinical characteristics of youths and parents seeking psychological treatment for school attendance problems
Source: PLoS One. 2022 Jan 26;17(1):e0261449. doi: 10.1371/journal.pone.0261449 (PMC8791456; doi:10.1371/journal.pone.0261449)
Supplement: S1 Table — (PDF) [file pone.0261449.s001.pdf]

S1 Table: *Mean and standard deviations from youth and parent ratings on all total scales and sub-scales of SCAS, MFQ, and SDQ grouped by gender and age group*

			Youths		Parents	
			Younger	*Older*	*Younger*	*Older*
			M (SD)	*M (SD)*	*M (SD)*	*M (SD)*
**SCAS-C**[a]	*Total*	Boys	33.70 (15.38)	22.76 (13.81)	36.43 (15.92)	25.76 (15.85)
		Girls	44.46 (18.41)	47.56 (17.36)	40.18 (13.87)	41.06 (18.65)
	Social phobia	Boys	6.80 (3.81)	5.61 (3.87)	8.98 (4.31)	7.15 (4.19)
		Girls	8.61 (5.03)	11.84 (3.81)	8.43 (4.53)	11.06 (4.13)
	Generalized anxiety	Boys	6.85 (3.62)	4.87 (3.59)	7.61 (3.57)	5.52 (3.36)
		Girls	8.79 (4.17)	9.66 (4.03)	8.21 (3.20)	8.81 (4.22)
	Obsessive-compulsive disorder	Boys	3.43 (2.23)	3.13 (2.84)	2.70 (2.50)	2.33 (2.61)
		Girls	4.39 (3.41)	5.47 (2.83)	3.25 (3.20)	3.28 (2.43)
	Panic disorder	Boys	5.20 (4.31)	3.15 (3.43)	4.35 (4.02)	3.22 (3.53)
		Girls	7.79 (5.51)	8.87 (6.03)	6.36 (4.54)	6.97 (6.29)
	Fear of physical injury	Boys	3.93 (2.82)	2.76 (2.31)	4.07 (2.86)	2.85 (2.57)
		Girls	5.04 (3.75)	5.53 (2.77)	4.57 (2.47)	3.94 (2.63)
	Separation anxiety	Boys	7.48 (4.34)	3.24 (2.93)	8.74 (4.53)	4.70 (4.00)
		Girls	9.86 (4.00)	6.19 (3.26)	9.36 (3.81)	7.00 (3.18)
**MFQ**[b]	*Total*	Boys	17.49 (11.29)	-	23.13 (12.86)	-
		Girls	27.22 (14.32)	-	27.68 (13.40)	-
**SDQ**[c]	*Total*	Boys	14.32 (6.00)	13.49 (5.87)	14.32 (5.77)	16.03 (5.83)
		Girls	15.73 (7.51)	16.10 (6.61)	16.09 (4.18)	16.14 (6.04)
	Conduct problems	Boys	2.23 (1.60)	2.09 (1.70)	2.14 (1.32)	2.39 (2.08)
		Girls	2.18 (1.40)	1.92 (1.87)	1.82 (1.66)	1.92 (1.93)
	Hyperactivity/ inattention	Boys	4.45 (2.74)	4.71 (2.58)	4.18 (2.68)	4.49 (2.49)
		Girls	3.73 (3.07)	4.47 (2.76)	3.91 (2.91)	3.92 (2.37)
	Peer relationship problems	Boys	2.00 (1.80)	2.21 (1.78)	1.95 (1.96)	3.14 (1.97)
		Girls	3.00 (1.67)	3.08 (2.05)	2.45 (1.13)	3.16 (2.07)
	Prosocial behavior	Boys	7.45 (1.41)	7.16 (1.94)	7.14 (2.46)	7.13 (2.18)
		Girls	8.00 (2.14)	7.84 (1.48)	7.55 (2.21)	7.67 (1.88)
	Emotional problems	Boys	5.64 (2.52)	4.47 (2.44)	6.05 (2.54)	6.01 (2.49)
		Girls	6.82 (3.28)	6.63 (2.46)	7.91 (1.97)	7.14 (2.35)
	Impact of problems	Boys	1.91 (1.80	1.96 (2.41)	4.45 (3.16)	4.79 (2.89)
		Girls	2.45 (2.50)	4.00 (3.14)	3.55 (2.66)	5.31 (3.10)

Note: SCAS: Spence Children's Anxiety Scale, MFQ: Mood and Feelings Questionnaire, SDQ-Total: Strength and Difficulties Questionnaire, M: Mean, SD: Standard Deviation,
[a]*Using age groups proposed by Arendt, et al., (2014): Children = 6 to 12 years, Adolescents = 13 to 17 years.*
[b]*No division of age group reported in Danish norms, all cases reported*
[c]*Using age groups proposed by Arnfred, et al., (2019): Children = 6 to 10 years, Adolescents = 11 to 17 years.*
